# Antibiotic-laden bone cement for diabetic foot infected wounds: A systematic review and meta-analysis

**DOI:** 10.3389/fendo.2023.1134318

**Published:** 2023-03-16

**Authors:** Tingting Dong, Qi Huang, Zengmei Sun

**Affiliations:** ^1^ Department of Endocrinology and Metabolism, Hospital of Chengdu Office of People’s Government of Tibetan Autonomous Region (Hospital.C.T.), Chengdu, China; ^2^ Department of Orthopedics, Hospital of Chengdu Office of People’s Government of Tibetan Autonomous Region (Hospital.C.T.), Chengdu, China

**Keywords:** bone cement, diabetic foot, infection, Antibiotics, meta-analysis, clinical efficacy

## Abstract

**Objective:**

A large body of literature has demonstrated the significant efficacy of antibiotic bone cement in treating infected diabetic foot wounds, but there is less corresponding evidence-based medical evidence. Therefore, this article provides a meta-analysis of the effectiveness of antibiotic bone cement in treating infected diabetic foot wounds to provide a reference basis for clinical treatment.

**Methods:**

PubMed, Embase, Cochrane library, Scoup, China Knowledge Network (CNKI), Wanfang database, and the ClinicalTrials.gov were searched, and the search time was from the establishment of the database to October 2022, and two investigators independently. Two investigators independently screened eligible studies, evaluated the quality of the literature using the Cochrane Evaluation Manual, and performed statistical analysis of the data using RevMan 5.3 software.

**Results:**

A total of nine randomized controlled studies (n=532) were included and, compared with the control group, antibiotic bone cement treatment reduced the time to wound healing (MD=-7.30 95% CI [-10.38, -4.23]), length of hospital stay (MD=-6.32, 95% CI [-10.15, -2.48]), time to bacterial conversion of the wound (MD=-5.15, 95% CI [-7.15,-2.19]), and the number of procedures (MD=-2.35, 95% CI [-3.68, -1.02]).

**Conclusion:**

Antibiotic bone cement has significant advantages over traditional treatment of diabetic foot wound infection and is worthy of clinical promotion and application.

**Systematic review registration:**

PROSPERO identifier, CDR 362293.

## Introduction

With the continuous improvement of socioeconomic and living standards, the global prevalence of diabetes is increasing rapidly and is expected to increase to 7.7% globally in 2030 (1). Diabetic foot infection (DFI) is one of the common complications of diabetic foot. The prevalence rate of diabetic foot is up to 25% (2, 3), of which the mortality rate is up to 12%. More than half of amputees are expected to die within 5 years. The mortality rate is higher than that of most cancers, posing a serious threat to patients’ health (4).

Current clinical practice guidelines on the treatment of DFIs guide essentially the same pattern of negative pressure closed drainage therapy, debridement and dressing changes, hematologic reconstruction, wound dressing, and education of patients and families (5–7). However, DFI is currently difficult to treat and the wound takes a long time to heal, often leading to extended hospital stays and increased hospital costs, adding to the patient’s burden.

As a unique bone repair material, antibiotic bone cement can release high concentrations of antibiotics locally to lower the risk of systemic toxicity and accomplish the goal of preventing and treating infection. According to studies, the use of antimicrobial bone cement in the treatment of DFI wounds has also yielded positive therapeutic outcomes. However, there is still no relevant evidence-based medical evidence to confirm this, so our article systematically evaluates the clinical efficacy of antibiotic bone cement in the treatment of diabetic foot to provide a reference basis for the future clinical treatment of diabetic foot.

## Methods

### Search strategy and selection criteria

This systematic review and meta-analysis are reported in accordance with the Preferred Reporting Items for Systematic Reviews and Meta-Analyses (PRISMA) (8) Statement and was registered in the International Prospective Register of Systematic Reviews (number CDR 362293).

The Embase, PubMed, Cochrane, Scoup, CNKI, CBM, and ClinicalTrials.gov were searched for relevant studies published between the database inception date and 11 October 2022. We applied no language restrictions. We used the following combined text and MeSH terms: “Diabetic Foot.” The complete search used for PubMed was: (“Diabetic Foot”[Mesh]) OR ((((Foot, Diabetic[Title/Abstract]) OR (Diabetic Feet[Title/Abstract])) OR (Feet, Diabetic[Title/Abstract])) OR (Foot Ulcer, Diabetic[Title/Abstract])). We considered all potentially eligible studies for review, irrespective of the primary outcome or language. We also conducted a manual search using the reference lists of key articles published in English (Supplementary materials).

### Study selection and data extraction

#### Inclusion criteria

(1) meeting the management of diabetic foot: a clinical practice guideline by the Society for Vascular Surgery in collaboration with the American Podiatric Medical Association and the Society for Vascular Medicine (9); (2) meeting the criteria for the diagnosis and treatment of diabetic foot infection published by the American Diabetes Association (ADA) infection (10); (3) the study is a randomized clinical trial.

#### Exclusion criteria

(1) Basic studies; (2) Conference reports and reviews; (3) Repeatedly published literature; (4) Literature with incomplete data; (5) Patients with non-diabetic foot infections; combined hematologic disorders other than anemia; and mental disorders were included in the literature.

### Screening and selection of literature

Two independent investigators (DTT, HJ) reviewed study titles and abstracts, and studies that satisfied the inclusion criteria were retrieved for full-text assessment. Trials selected for detailed analysis and data extraction were analyzed by two investigators (DTT and HJ); disagreements were resolved by a third investigator (SZM).

We extracted the following data from each selected study: total number of participants, age, sex, trial duration, and treatment modality. Two independent reviewers (DTT, HJ) assessed the risk of bias according to the PRISMA recommendations.

### Outcome indicators

The main outcome indicators were wound healing time, length of hospital stay, time for bacterial culture Negative-conversing, and number of surgeries.

### Patient and public involvement

Patients and/or the public were not involved in the design, conduct, reporting, or dissemination plan of this study.

### Quality evaluation of included studies

The quality of the included articles was assessed by the Cochrane RoB 2.0 tool (11, 12), which included the following: (1) risk of bias arising from the randomization process; (2) risk of bias due to deviations from the intended interventions (effect of assignment to intervention); (3) missing outcome data; (4) risk of bias in the measurement of the outcome; and (5) risk of bias in the selection of the reported result. Each study was assessed as having a “low risk of bias,” “some concerns,” or “high risk of bias” according to the probability of bias.

### Data synthesis

All data processing and statistical analyses were performed using Review Manager (RevMan) software version 5.3 (http://www.ims.cochrane.org/revman/). Heterogeneity between studies was evaluated by the Q statistic and the I (2) statistic. When I (2) < 50%, p > 0.05, indicating little or no heterogeneity, a fixed effects model was used; when I (2) > 50%, p < 0.05, indicating significant heterogeneity, a random effects model was used. Subgroup analysis was also conducted, with a sample size cut-off of n=50, to compare the combined effect results of small and large sample subgroups and to explore sources of heterogeneity. Publication bias was assessed using funnel plots, sensitivity analyses were conducted using the sequential omission of a single study from the total studies and evaluating the influence of each study on the pooled effect estimates, and subgroup analyses were conducted based on the general characteristics of the study population and sample size.

## Results

### Screening and selection of literature

**Figure 1:**
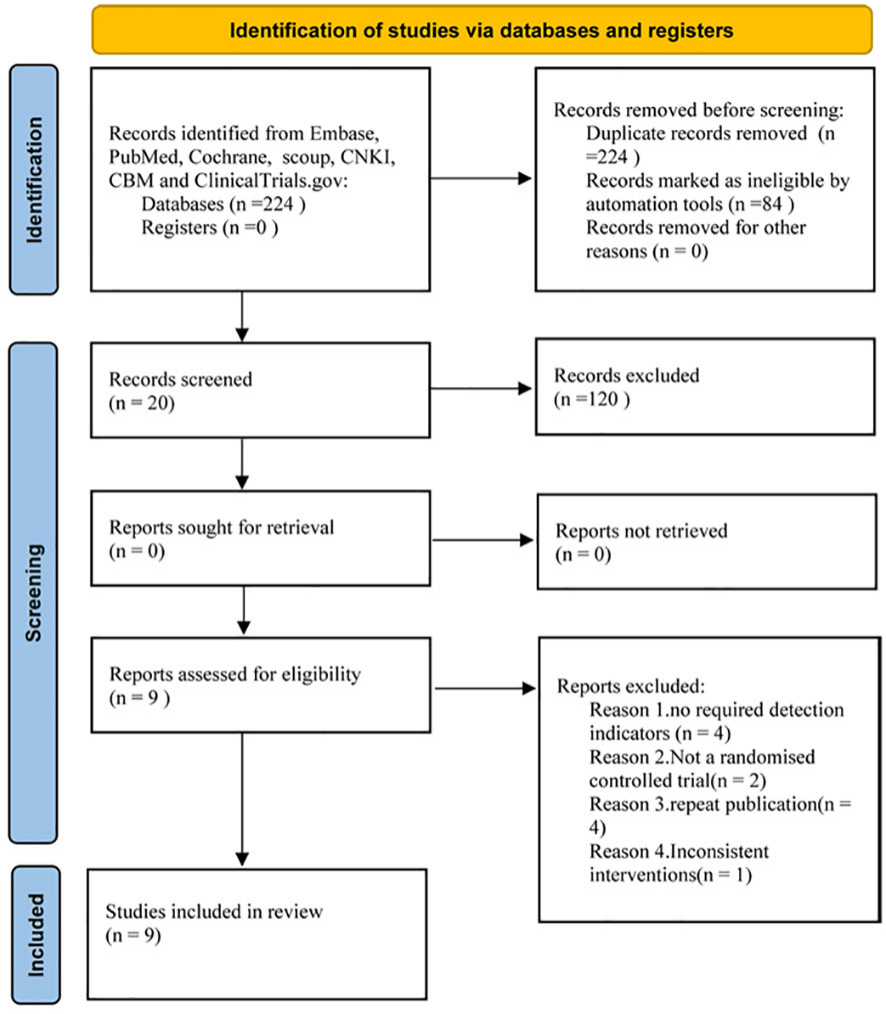
Flowchart of the meta-analysis.

We identified 224 studies, downloaded the full texts to read, and excluded 11, of which, nine (with data for 532 participants) were included in our analysis (**Figure 1**), all of which were randomized controlled studies. The nine trials (13–21)were all published between 2016 and 2022.

### Design characteristics

**Figure 2:**
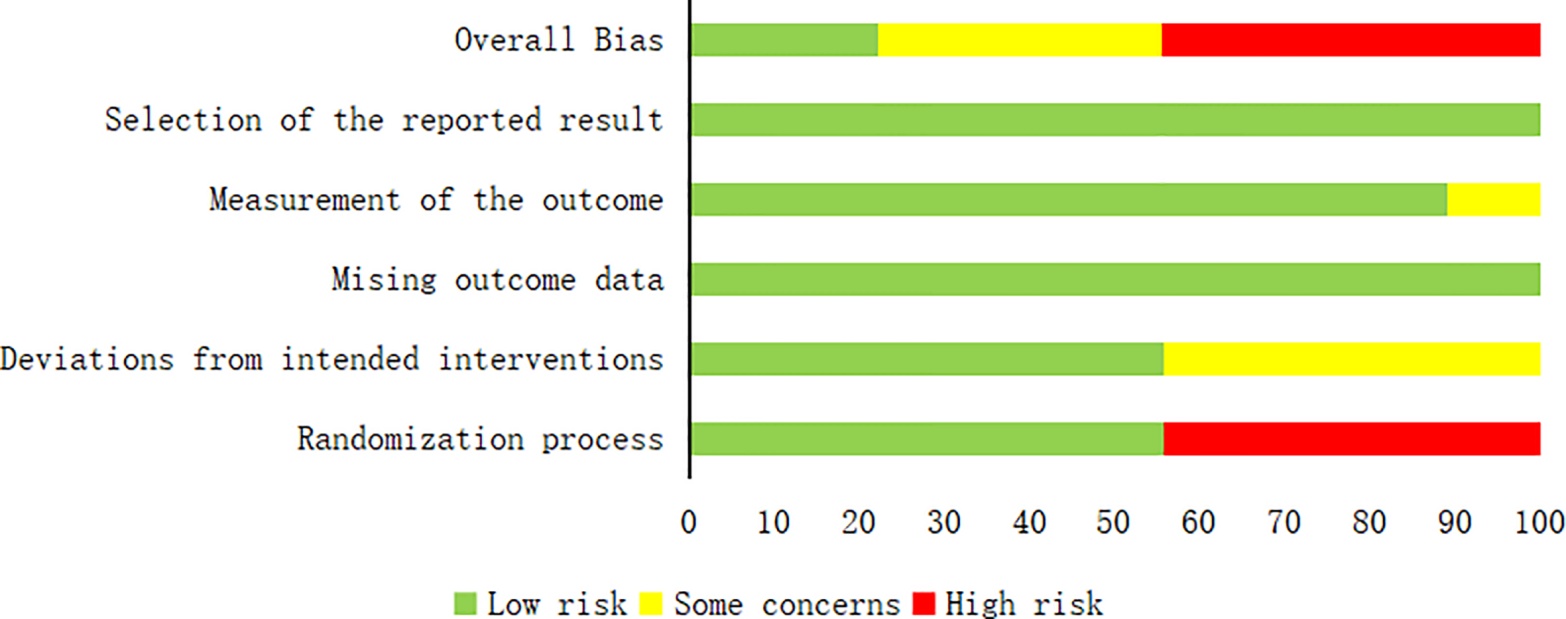
Risk of bias graph.

**Figure 3:**
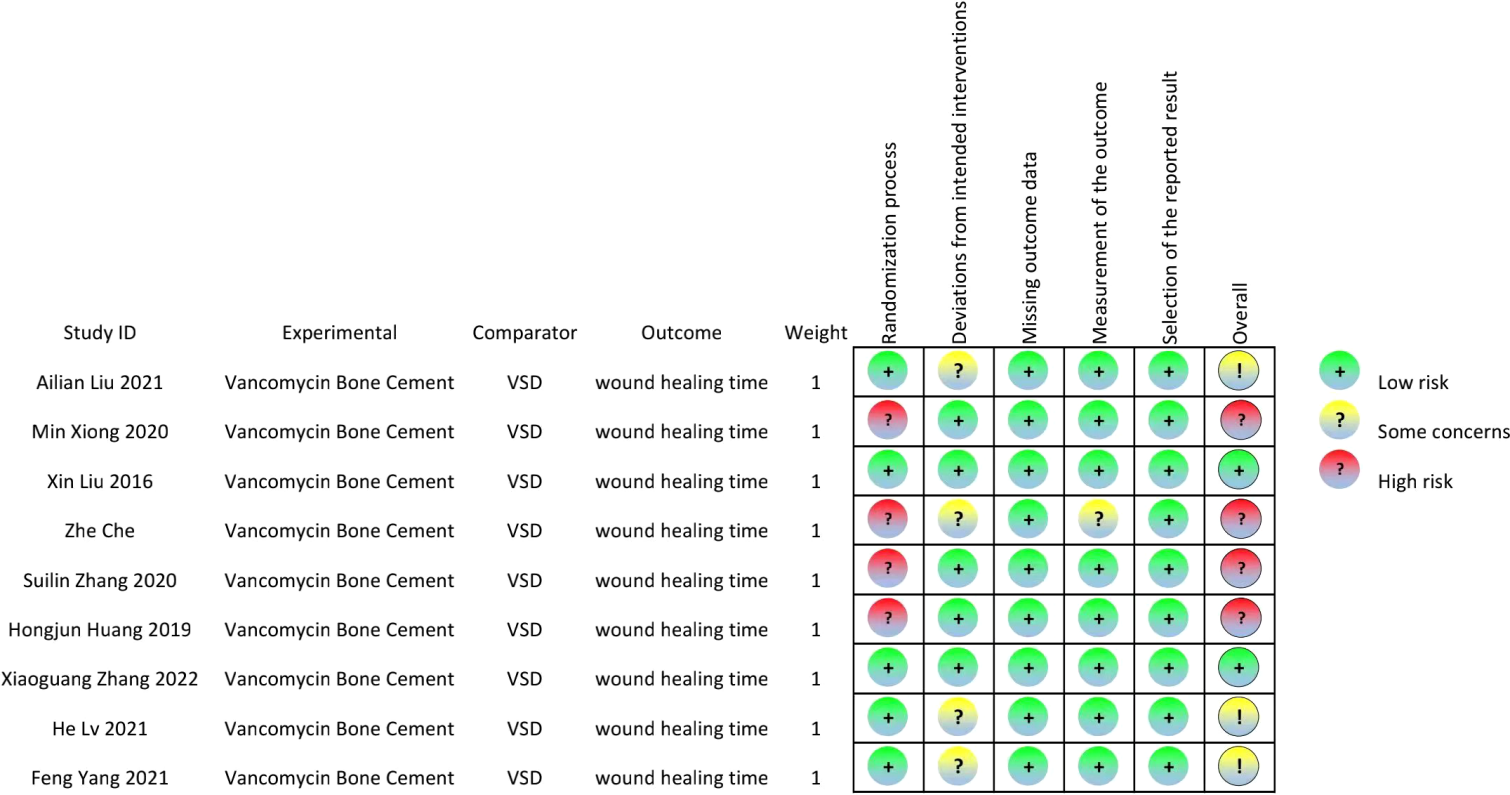
Risk of bias summary.

A total of 532 patients with diabetic foot were included in nine randomized controlled trials (13–21), with 266 patients in the intervention group and 266 in the control group. The subjects included in the study were aged 31-88 years. All had diabetic foot with a Wagner classification of 1-5. In the intervention group, cefoperazone bone cement was used in 13 patients, gentamicin bone cement in six patients, and vancomycin bone cement in 27 patients in the study by Xin Liu et al.^[13]^. The remaining eight studies were treated with vancomycin bone cement and the control group was treated with VSD. The baseline characteristics of the study participants and design characteristics are presented in **Table 1**. The quality of the literature is assessed in **Figures 2**, **3**.

**Table 1:** General characteristics of included literature.

Name	Year	Parallel design	Age	Number of patients	Man/ female	Treatment modality		Intervention time (day)	Wagner classification for diabetic foot	Diabetic foot wound area	Diabetic foot duration (week)	Observed indicators
						Control group	Test group					
Ailian Liu (13)	2021	Yes	43-75	66	39/27	VSD	Vancomycin Bone Cement	N/A	NR	10-75	1-6	1
Min Xiong (14)	2020	Yes	41-88	80	37/43	VSD	Vancomycin Bone Cement	47	2-4	8-40	N/A	1,2,3,4
Xin Liu (15)	2016	Yes	35-72	46	23/23	VSD	Vancomycin Bone Cement; Cefoperazone Bone Cement; Gentamicin Bone Cement	56	2-4	8-20	4.63-6.63	1,2,4
Zhe Chen (16)	2022	Yes	46-78	90	48/42	VSD	Vancomycin Bone Cement	38	3-5	N/A	30-102.86	2,3,4
Suling Zhang (17)	2020	Yes	51.6-82	60	44/16	VSD	Vancomycin Bone Cement	84	>2	N/A	N/A	1
Hongjun Huang (18)	2019	Yes	53-79	36	N/A	VSD	Vancomycin Bone Cement	39	3-4	6-77	1-5.86	1,2,4
Xiaoguang Zhang (19)	2022	Yes	41-83	88	45/43	VSD	Vancomycin Bone Cement	15	1-4	5-35	52.14-156.43	1,2,3,4
He Lv (20)	2021	Yes	66-81	32	17/15	VSD	Vancomycin Bone Cement	41	2-4	N/A	48.51-159.92	1,2,3,4
Feng Yang (21)	2021	Yes	31-84	34	N/A	VSD	Vancomycin Bone Cement	84	2-4	N/A	N/A	1

VSD, vacuum sealing drainage; Observed indicators: 1, wound healing time; 2, length of hospital stay; 3, Time for bacterial culture Negative-conversing; 4, number of surgeries; N/A means not reported.

### Meta-analysis results

#### Wound healing time

**Figure 4:**
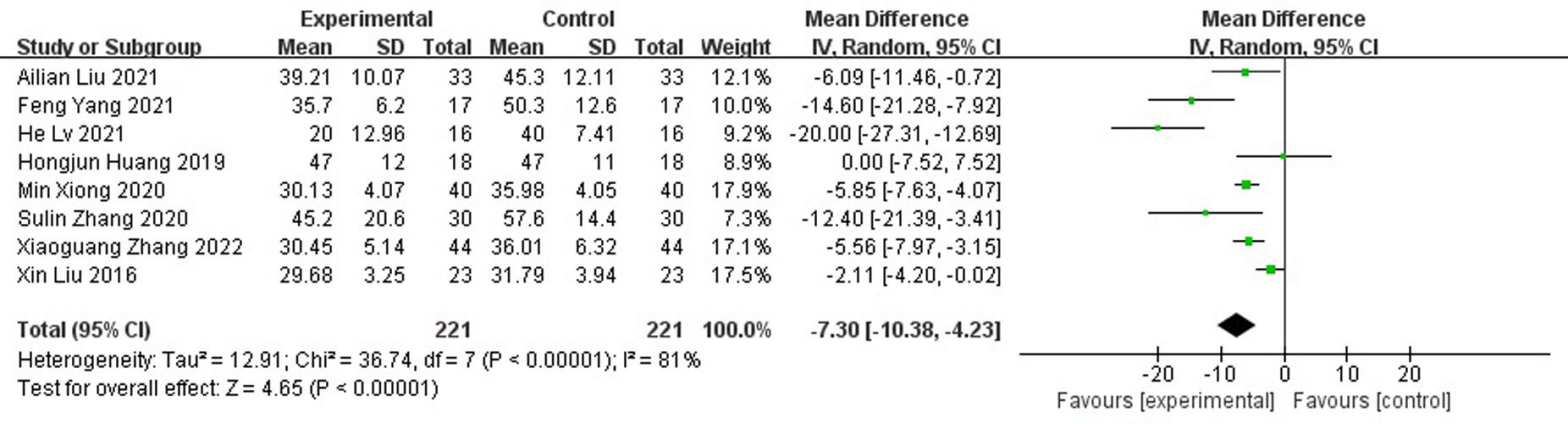
Forest plot of wound healing time.

**Figure 5:**
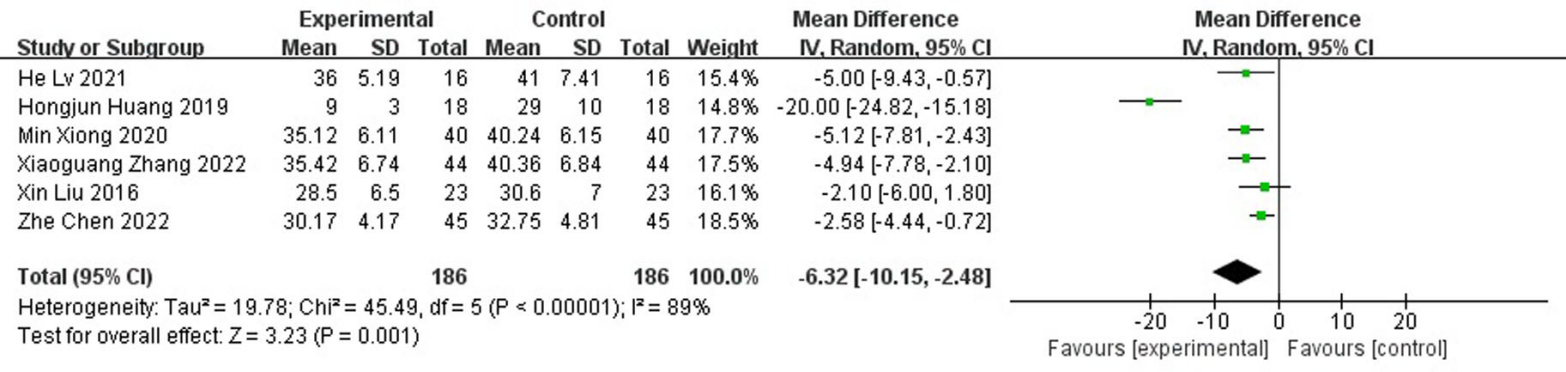
Subgroup analysis of wound healing time.

Compared with those of the control group, wound healing time was shortened (MD=-7.30 95% CI[-10.38, -4.23]), As the meta-analysis showed a high degree of heterogeneity with Tau^2 =^ 12.91, I^2 =^ 81%, p<0.001, a random effects model was used for the combined analysis (**Figure 4**). Subgroup analysis according to sample size showed significantly lower heterogeneity in both the subgroup with sample size < 50 (-8.95[-17.98,0.09], I^2 =^ 91%, p<0.001) and the subgroup with sample size ≥ 50 (-5.92[-7.29,-4.56], I^2 =^ 0%, p=0.52). The antibiotic bone cement treatment of diabetic foot was shorter than the control group in terms of wound healing time in all cases (**Figure 5**).

#### Length of hospital stay

**Figure 6:**
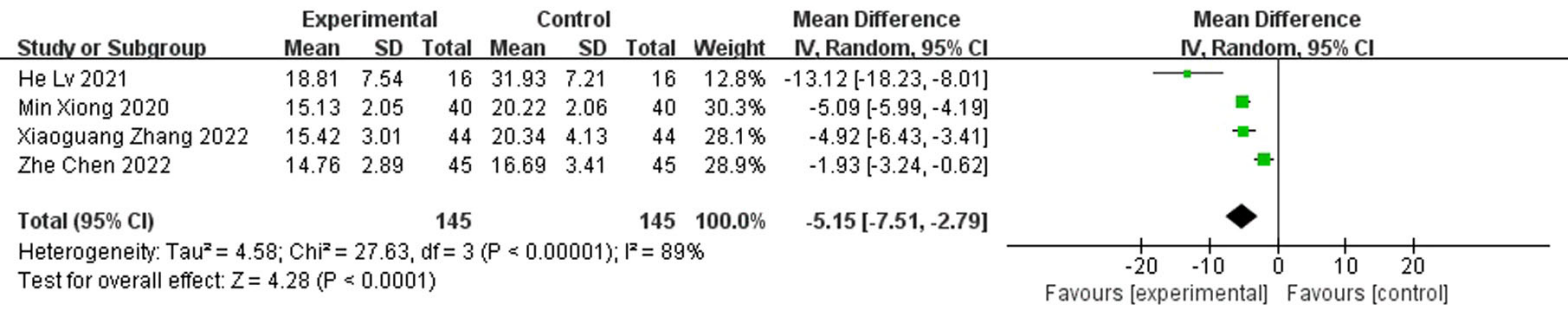
Forest plot of length of hospital stay.

**Figure 7:**
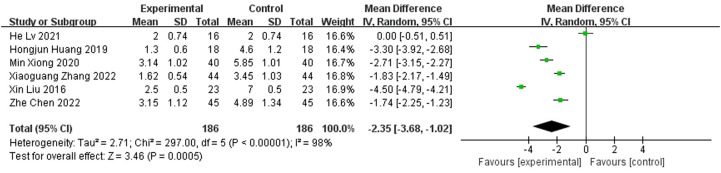
Subgroup analysis of length of hospital stay.

A total of six papers reported the length of hospital stay and the results showed that the length of hospital stay was shorter for antibiotic bone cement for diabetic foot than the control group (MD=-6.32, 95% CI [-10.15,-2.48]) and there was heterogeneity in the data (Tau^2 =^ 19.78, I^2 =^ 89%, P < 0.001) (**Figure 6**). Subgroup analysis according to the sample size showed significantly lower heterogeneity in both the subgroup with sample size <50 (-8.96[-19.42,1.49], I2 = 94%, p<0.001) and the subgroup with sample size ≥ 50 (-6.32[-10.15,-2.48], I2 = 37%, p=0.2). The length of stay was shorter for antibiotic bone cement for diabetic foot than the control group (**Figure 7**).

#### Time for bacterial culture negative-conversing

**Figure 8:**
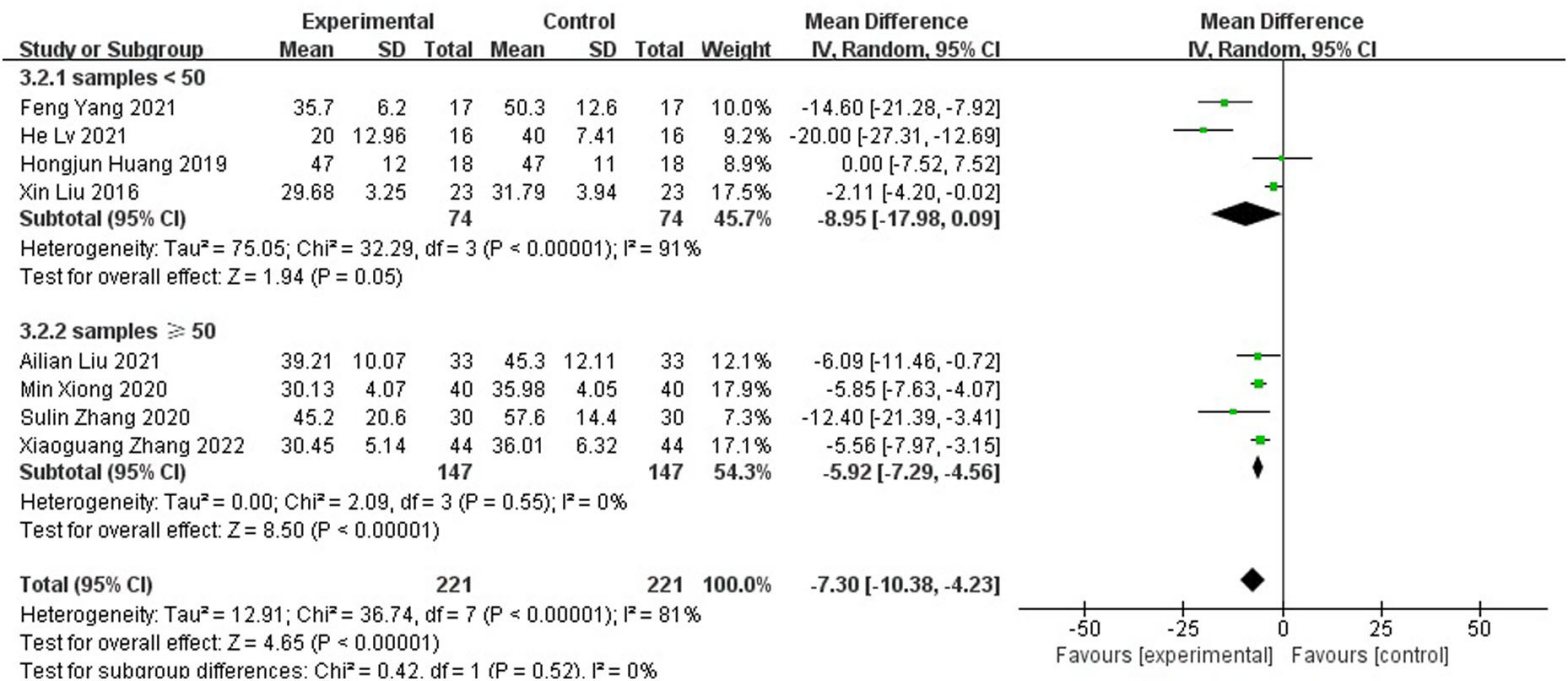
Forest plot of Time for bacterial culture Negative-conversing.

A total of four papers reported bacterial culture turnaround times, which showed shorter bacterial culture turnaround times in diabetic foot treated with antibiotic bone cement than in the control group (MD=-5.15,95% CI [-7.15,-2.19]) and there was heterogeneity in the data (Tau^2 =^ 4.58, I^2 =^ 89%, P < 0.001); therefore, a random effects model was used to calculate the combined effect size (**Figure 8**).

### Number of surgeries

**Figure 9:**
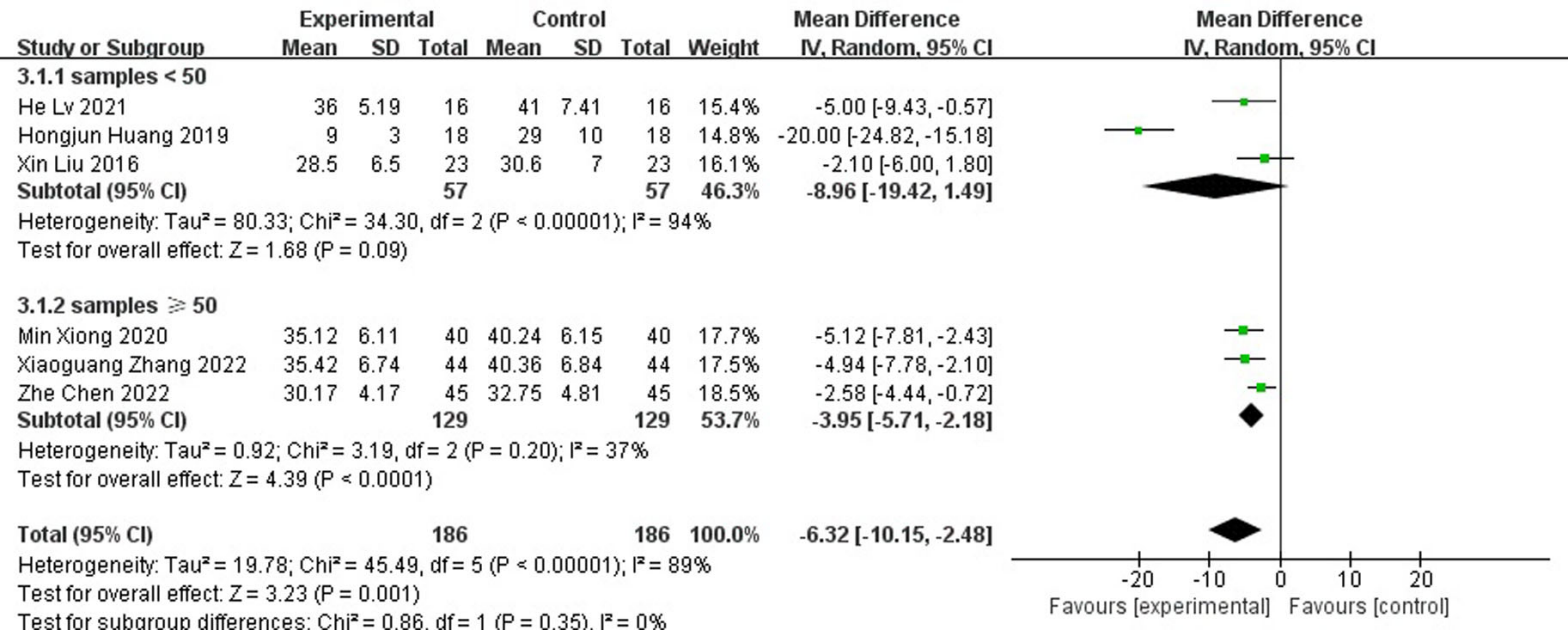
Forest plot of number of surgeries.

A total of six publications reported the number of procedures and showed that the number of procedures for diabetic foot treated with antibiotic bone cement was less than the control group (MD=-2.35,95% CI [-3.68, -1.02]) and there was heterogeneity in the data (Tau^2 =^ 2.71, I^2 =^ 89%, P < 0.001); therefore, a random effects model was used to calculate the combined effect size (**Figure 9**).

## Discussion

Our results show that antibiotic bone cement treatment reduced wound healing time, length of hospital stay, time to negative bacterial culture of wound secretions, and the number of procedures compared with other diabetic foot treatments, and the results provide support for antibiotic bone cement as a treatment for diabetic foot infections.

Patients with DFI have low resistance and skin tissue regeneration capacity, resulting in slow wound healing, and these open wounds are susceptible to invasion by pathogenic bacteria, resulting in serious infections (22). Patients with DFI are treated with systemic antibiotics against infection, but the long-term application of antibiotics will produce adverse effects and bacterial resistance (23). Sun Shujuan et al. (24) investigated and analyzed the pathogenic bacteria of diabetic foot in Beijing and showed that multi-drug-resistant bacteria accounted for 16.9%. Due to the long trauma healing time, long hospital stay, and high medical costs of DFI patients, it not only imposes a heavy burden on clinical care but also causes increased anxiety and depression in patients, which affects treatment outcomes and quality of life (25, 26). In addition, routine debridement and surgical treatment for DFI may cause improper wound management, resulting in longer healing times, easy recurrence of ulcers, increased risk of metastatic ulcers, amputation, and death (27, 28).

As shown in **Figures 4**, **6**, antibiotic bone cement for DFI reduced wound healing time and length of hospital stay compared to the controls, which is consistent with other results reported in the literature (13, 29). This may be due to the fact that when the bone cement covers the wound, it produces a 1-2 mm thick biofilm, which becomes an induced membrane and is capable of secreting relevant cytokines to promote wound healing, such as transforming growth factor-β1 (TGF-β1), vascular endothelial growth factor (VEGF), etc. The secreted cytokines also have angiogenic and potential osteogenic properties (30–32). Most patients with DFI have pathological manifestations of small blood vessels and capillary blockages at the ends of the limbs, and the formation of IM promotes fresh angiogenesis, which, in turn, improves blood flow to the extremities, facilitating wound healing and shortening the patient’s hospital stay (33–37).

Our meta-analysis research showed that antibiotic bone cement treatment shortened the bacterial turnaround time of the trauma and reduced the number of procedures compared with conventional debridement combined with negative pressure closure and drainage treatment. Due to poor blood flow to the foot and reduced peripheral perfusion in patients with DFI, intravenous systemic antibiotics reduce delivery to bone or soft tissues and limit their efficacy, thus not achieving effective bactericidal concentrations at the site of the lesion. Antibiotic bone cement has eluting properties, releasing a much higher concentration of antibiotics than systemically applied antibiotics, with an efficiency of 81%, effectively preventing the emergence of drug-resistant strains (38). The bone cement gradually creates a local sterile environment with a slow and continuous local release of antibiotics, which can act directly on the lesion area and, thus, kill the bacteria, shortening the time until bacterial transformation of the wound (39, 40). In addition, the drug released locally rarely enters the systemic circulatory system, thus reducing the side effects of antibiotics (41). The simultaneous application of antibiotic bone cement treatment is easy to perform, with short operative times, easy post-operative care and dressing changes, and lower overall treatment costs.

Vancomycin is a common additive to antibiotic bone cement, releasing topical vancomycin at a concentration of approximately 0.5-2.0 μg/mL to meet the minimum inhibitory concentration requirements (42). In assessing the breadth of response to the dynamic release of vancomycin, it was found that the plateau period for vancomycin release could be >10 days (43–45). Vancomycin has a broad spectrum of sensitive bacteria and kills the most common pathogenic microorganisms (46). In the intervention group, the wound was covered with vancomycin using bone cement as a carrier, and the infection was effectively controlled, facilitating wound healing.

Our study also has some shortcomings. First, the sample size of the included literature was small, with four papers having a sample size of fewer than 50 cases. Second, the different lengths of interventions and inconsistent outcome indicators in the included literature may cause some bias in the study results and increase the heterogeneity of the Meta-analysis results. Finally, the number of high-quality literature included is limited, and more multicenter, large sample randomized controlled clinical trials are needed.

In summary, antibiotic bone cement is effective in treating DFI wounds, saving medical resources and costs, and it is worth promoting its use in clinical practice. In the future, more multicenter and large sample size randomized controlled clinical trials should be conducted to increase the in-depth discussion on the induction of film formation by trauma bone cement in different periods of the diabetic foot and the most suitable types of trauma for antibiotic bone cement, to comprehensively evaluate the effectiveness of antibiotic bone cement in the treatment of diabetic foot and provide a more rigorous and objective reference basis for the treatment of diabetic foot in the clinic in the future.

## Data availability statement

The original contributions presented in the study are included in the article/supplementary material. Further inquiries can be directed to the corresponding author.

## Author contributions

TD and QH contributed equally to the design of the study. TD, QH, and ZS collected the data and performed the qualitative analysis. QH performed the quantitative analysis. TD, QH, and ZS drafted the manuscript. TD, QH, and ZS participated in the discussion and interpretation of the data. All authors approved this final version for publication. ZS acted as guarantor, assumed full responsibility for the completion of the article, had access to all data, and controlled the decision to publish.
